# Supplementary material for: BioLACE: unifying spatial geometry and marker priors for cohesive cell-type clustering in spatial transcriptomics
Source: bioRxiv. 2025 Dec 2:2025.12.01.691603. Preprint. [Version 1] doi: 10.64898/2025.12.01.691603 (PMC12822702; doi:10.64898/2025.12.01.691603)
Supplement: Supplementary file 1 [file NIHPP2025.12.01.691603v1-supplement-1.pdf]

bioRxiv preprint doi: https://doi.org/10.64898/2025.12.01.691603; this version posted December 2, 2025. The copyright holder for this preprint (which was not certified by peer review) is the author/funder, who has granted bioRxiv a license to display the preprint in perpetuity. It is made available under a CC-BY 4.0 International license.

Supplementary Methods

1. Expanded ablation details: MERFISH hypothalamus

Motivation. To isolate the contribution of each objective in BioLACE, we ablated the VAE backbone, Laplacian regularization, and marker-aware contrastive term on the MERFISH mouse hypothalamus dataset. The hypothalamus combines compact neuronal nuclei with diffuse glial regions, providing a stringent test of how spatial and biological constraints interact.

Stepwise model development. Supplementary Fig. 3 details the effect of each added regularization term in BioLACE, and Supplementary Table 3 details the quantitative analysis and insights. We began with a *vanilla VAE* (ARI 0.42), which captures global transcriptomic structure but lacks geometric awareness, missing small spatially restricted groups. Adding a *contrastive loss* alone maintained comparable performance (ARI 0.41) with slightly sharper boundaries but unstable partitions—biological similarity alone proved insufficient without spatial context. Introducing the *graph Laplacian* (VAE + Laplacian) improved ARI to 0.45 by promoting local smoothness and alignment with tissue neighborhoods but over-smoothed boundaries between adjacent yet distinct types. Combining both spatial and contrastive objectives required temperature tuning: low τ=0.1 over-segmented (ARI 0.40), high τ=1.0 over-smoothed (ARI 0.47). At τ=0.5, the full model achieved ARI 0.62, cleanly recovering compact neuronal domains.

Interpretation. The VAE provides a denoised latent manifold; the Laplacian preserves local topology; and the marker-aware contrastive term sharpens biologically meaningful axes. Calibrated jointly, these components resolve the smoothness–separability trade-off: spatial alignment alone merges types, contrastive separation alone fragments gradients, but the combined objective yields anatomically faithful, biologically interpretable clusters.

bioRxiv preprint doi: https://doi.org/10.64898/2025.12.01.691603; this version posted December 2, 2025. The copyright holder for this preprint (which was not certified by peer review) is the author/funder, who has granted bioRxiv a license to display the preprint in perpetuity. It is made available under a CC-BY 4.0 International license.

2. Evaluation Metrics for Cell Clustering and Spatial Analysis

Clustering concordance metrics

Adjusted Rand Index (ARI). Measures agreement between predicted clusters and reference labels by counting pairwise co-assignments, with a correction for chance. Given two partitions of N samples—predicted clusters $\{C_k\}$ and reference labels $\{L_\ell\}$—with contingency table entries $n_{k\ell} = |C_k \cap L_\ell|$, ARI is:

$$\text{ARI} = \frac{\sum_{k,\ell}\binom{n_{k\ell}}{2} - \frac{[\sum_k \binom{|C_k|}{2}][\sum_\ell \binom{|L_\ell|}{2}]}{\binom{N}{2}}}{\frac{1}{2}\Big[\sum_k \binom{|C_k|}{2} + \sum_\ell \binom{|L_\ell|}{2}\Big] - \frac{[\sum_k \binom{|C_k|}{2}][\sum_\ell \binom{|L_\ell|}{2}]}{\binom{N}{2}}}.$$

Values range from 0 (chance) to 1 (perfect), with negative values indicating worse-than-random alignment.

V-measure. Evaluates cluster quality as the harmonic mean of homogeneity (each predicted cluster contains primarily one true label) and completeness (each true label maps to a single predicted cluster). Let $H(\cdot)$ denote entropy:

$$h = 1 - \frac{H(L \mid C)}{H(L)}, \qquad c = 1 - \frac{H(C \mid L)}{H(C)}, \qquad \text{V-measure} = 2 \cdot \frac{h\,c}{h + c}.$$

Spatial autocorrelation metric

Moran's I. Quantifies how strongly gene expression values co-vary among spatial neighbors, detecting whether high or low expression forms spatially contiguous domains. Given expression $\mathbf{g}$ and spatial weight matrix W:

$$I = \frac{N}{\sum_{i\neq j} w_{ij}} \cdot \frac{\sum_{i\neq j} w_{ij}(g_i - \bar{g})(g_j - \bar{g})}{\sum_i (g_i - \bar{g})^2}.$$

Values near 1 indicate strong spatial clustering.

Gene–gene coherence

Spearman's ρ. Measures monotonic co-expression between two genes by comparing rank-order agreement:

$$\rho = 1 - \frac{6\sum_i d_i^2}{m(m^2 - 1)},$$

where d_i is the difference in ranks.

Binary classification performance

Accuracy. Fraction of correctly labeled samples:

$$\text{Accuracy} = \frac{1}{N}\sum_{i=1}^{N} \mathbf{1}[\hat{y}_i = y_i].$$

Recall. Fraction of true positive samples recovered:

$$\text{Recall} = \frac{\text{TP}}{\text{TP} + \text{FN}}.$$

AUROC. Probability that a randomly chosen positive example is assigned a higher score than a negative:

$$\text{AUROC} = \Pr(s_i > s_j \mid y_i = 1,\, y_j = 0).$$

Segmentation and spatial-overlap metric

Dice Coefficient. Measures spatial overlap between predicted and reference regions:

$$\text{Dice} = \frac{2|A \cap B|}{|A| + |B|}.$$

Higher values indicate tighter anatomical correspondence.

bioRxiv preprint doi: https://doi.org/10.64898/2025.12.01.691603; this version posted December 2, 2025. The copyright holder for this preprint (which was not certified by peer review) is the author/funder, who has granted bioRxiv a license to display the preprint in perpetuity. It is made available under a CC-BY 4.0 International license.

Supplementary Tables

Supplementary Table 1: Slide-seq summary: spatial and functional validation under BioLACE. Metrics use the best BioLACE cluster for each domain.

Dataset	Domain (cluster)	Markers	Moran's I	AUROC	Within ρ	Dice
V1	Purkinje (4)	*Pcp4*, *Pcp2*	0.263	0.948	0.649	0.699
V1	Myelin (1)	*Plp1*, *Mbp*	0.289	0.839	0.200	0.678
V2	Purkinje (8/2)	*Pcp2*, *Pcp4*	0.302	0.812	0.709	0.506
V2	Myelin (5)	*Plp1*, *Mbp*	0.319	0.968	0.469	0.728

Supplementary Table 2: Curated marker genes and rationale for inclusion. Each group represents a biologically distinct program or anatomical niche in the spinal cord.

Category	Representative markers	Rationale / expected cell type
Excitatory neurons	*Slc17a8*, *Tac2*, *Nmu*, *Prkcg*, *Maf*, *Rorb*	Dorsal horn excitatory subtypes involved in sensory and interneuron circuits
Inhibitory neurons	*Pdyn*, *Npy*, *Npy2r*, *Cdh3*, *Sox5*	Inhibitory microcircuits in dorsal and medial laminae
Motoneurons	*Chat*, *Slc18a3*	α and γ motor pools in the ventral horn
Glial / myelinating cells	*Pdgfra*, *Opalin*, *Spp1*, *Agt*	Oligodendrocyte progenitors and astrocytic support cells
Vascular / pericyte	*Abcc9*, *Flt1*, *Eng*, *Notch3*, *Col25a1*	Endothelial and perivascular populations maintaining blood–spinal-barrier function
Immune / microglia	*Aif1*, *Trem2*, *C3*, *P2ry13*	Resident microglia and complement-activated immune states
Ependymal / ciliated	*Ddr1*, *Pkd2l1*, *Cfap43*	Central canal and cerebrospinal-fluid–contacting ependymal cells

bioRxiv preprint doi: https://doi.org/10.64898/2025.12.01.691603; this version posted December 2, 2025. The copyright holder for this preprint (which was not certified by peer review) is the author/funder, who has granted bioRxiv a license to display the preprint in perpetuity. It is made available under a CC-BY 4.0 International license.

Supplementary Table 3: Ablation study on MERFISH hypothalamus. Each component contributes uniquely to clustering performance; only the joint model achieves both spatial and biological coherence.

Model Variant	ARI	Temperature	Observation / Remark
VAE only	0.42	–	Captures global transcriptomic structure but fails to resolve small or spatially localized clusters.
VAE + contrastive loss	0.41	–	Slightly sharper boundaries but lacks spatial coherence and stable partitions.
VAE + spatial Laplacian	0.45	–	Improves local smoothness but merges adjacent cell types; lacks discriminative power.
VAE + Laplacian + contrastive (τ=1.0)	0.47	high	Contrastive gradients too weak; clusters over-smooth and lose separation.
VAE + Laplacian + contrastive (τ=0.1)	0.40	low	Over-segmentation and unstable boundaries due to excessive contrastive strength.
VAE + Laplacian + contrastive (τ=0.5)	**0.62**	tuned	Best overall balance: spatial continuity *and* biological separability; recovers fine neuronal clusters cleanly.

bioRxiv preprint doi: https://doi.org/10.64898/2025.12.01.691603; this version posted December 2, 2025. The copyright holder for this preprint (which was not certified by peer review) is the author/funder, who has granted bioRxiv a license to display the preprint in perpetuity. It is made available under a CC-BY 4.0 International license.

Supplementary Figures

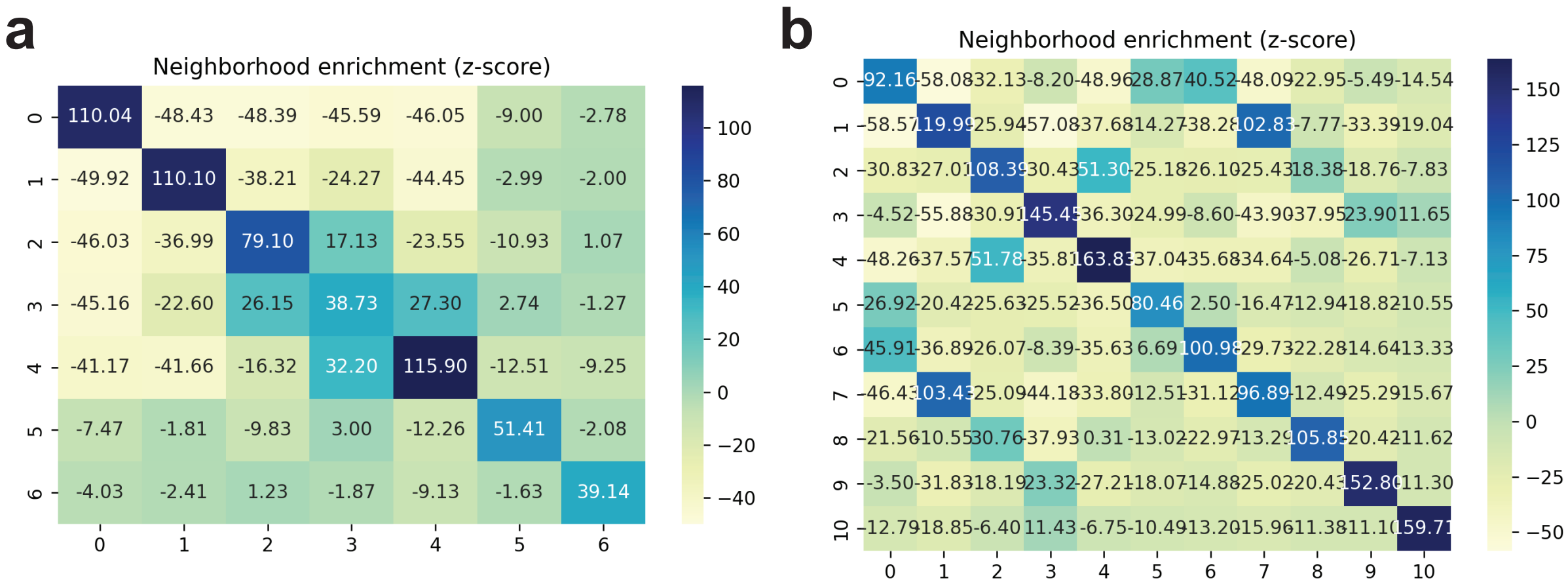

Supplementary Fig. 1: Neighborhood coherence under BioLACE (Slide-seq V1/V2). SQUIDPY neighbor-enrichment matrices for **(a)** V1 and **(b)** V2. BioLACE shows consistently higher same-label probability among spatial neighbors compared with baselines, indicating that Laplacian alignment preserves local tissue topology without oversmoothing across known boundaries.

bioRxiv preprint doi: https://doi.org/10.64898/2025.12.01.691603; this version posted December 2, 2025. The copyright holder for this preprint (which was not certified by peer review) is the author/funder, who has granted bioRxiv a license to display the preprint in perpetuity. It is made available under a CC-BY 4.0 International license.

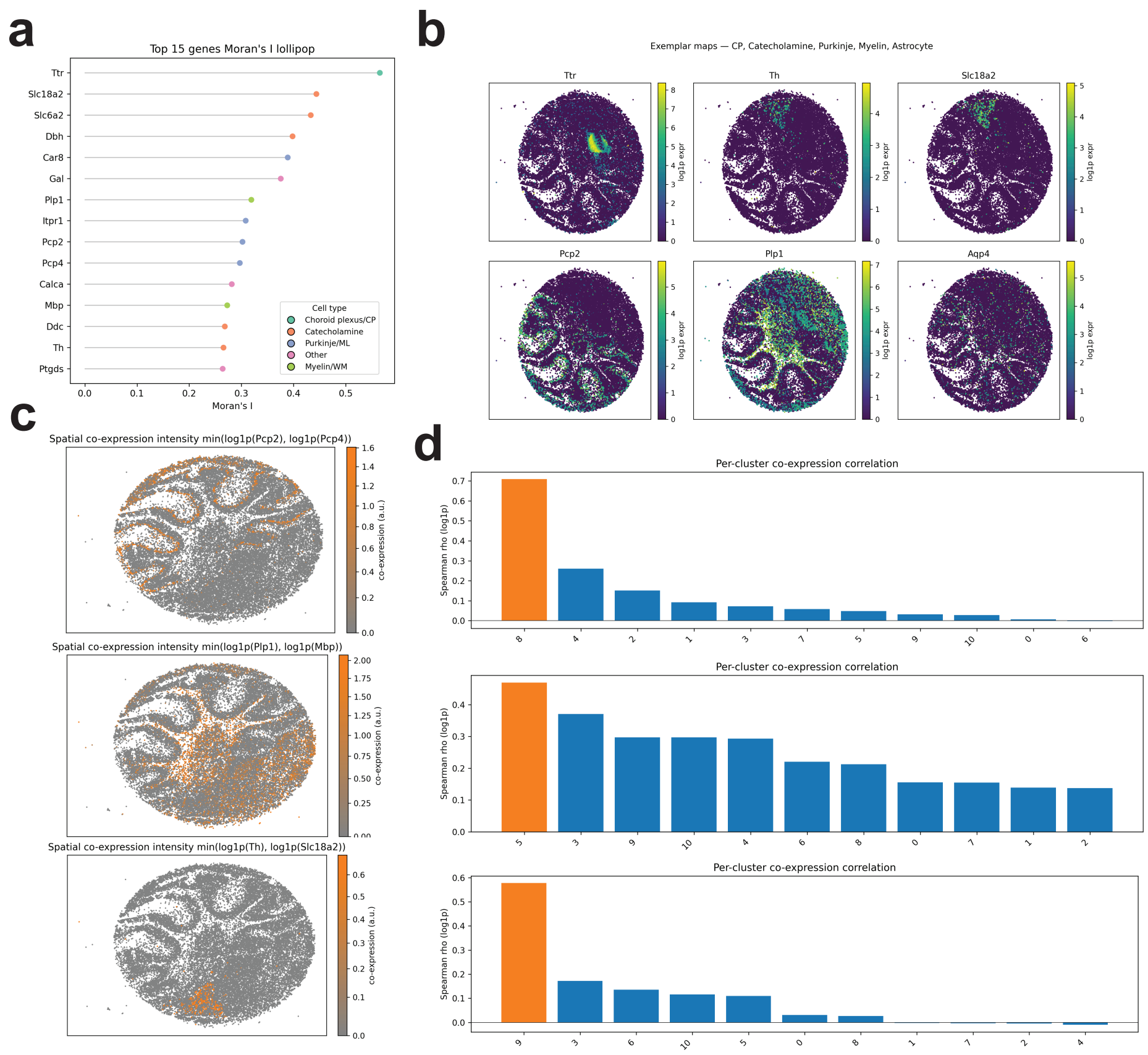

Supplementary Fig. 2: Slide-seq V2—Moran's I, co-expression, and cross-method comparison. **(a)** Lollipop plot of Moran's I identifies top spatially autocorrelated markers: *Plp1* (myelin, I=0.319), *Pcp2* (Purkinje, I=0.302), and *Ttr* (ependymal/CSF, I=0.565). **(b)** Spatial overlays show distinct localization of myelin and Purkinje domains, recapitulating cerebellar architecture. **(c)** Within-cluster co-expression peaks coincide with Moran's I maxima: *Pcp2*–*Pcp4* ρ=0.709 (Purkinje) and *Plp1*–*Mbp* ρ=0.469 (myelin). **(d)** BioLACE reveals a cluster coexpressing *Th* and *Slc18a2* that is not recovered by RCTD, illustrating added biological resolution.

bioRxiv preprint doi: https://doi.org/10.64898/2025.12.01.691603; this version posted December 2, 2025. The copyright holder for this preprint (which was not certified by peer review) is the author/funder, who has granted bioRxiv a license to display the preprint in perpetuity. It is made available under a CC-BY 4.0 International license.

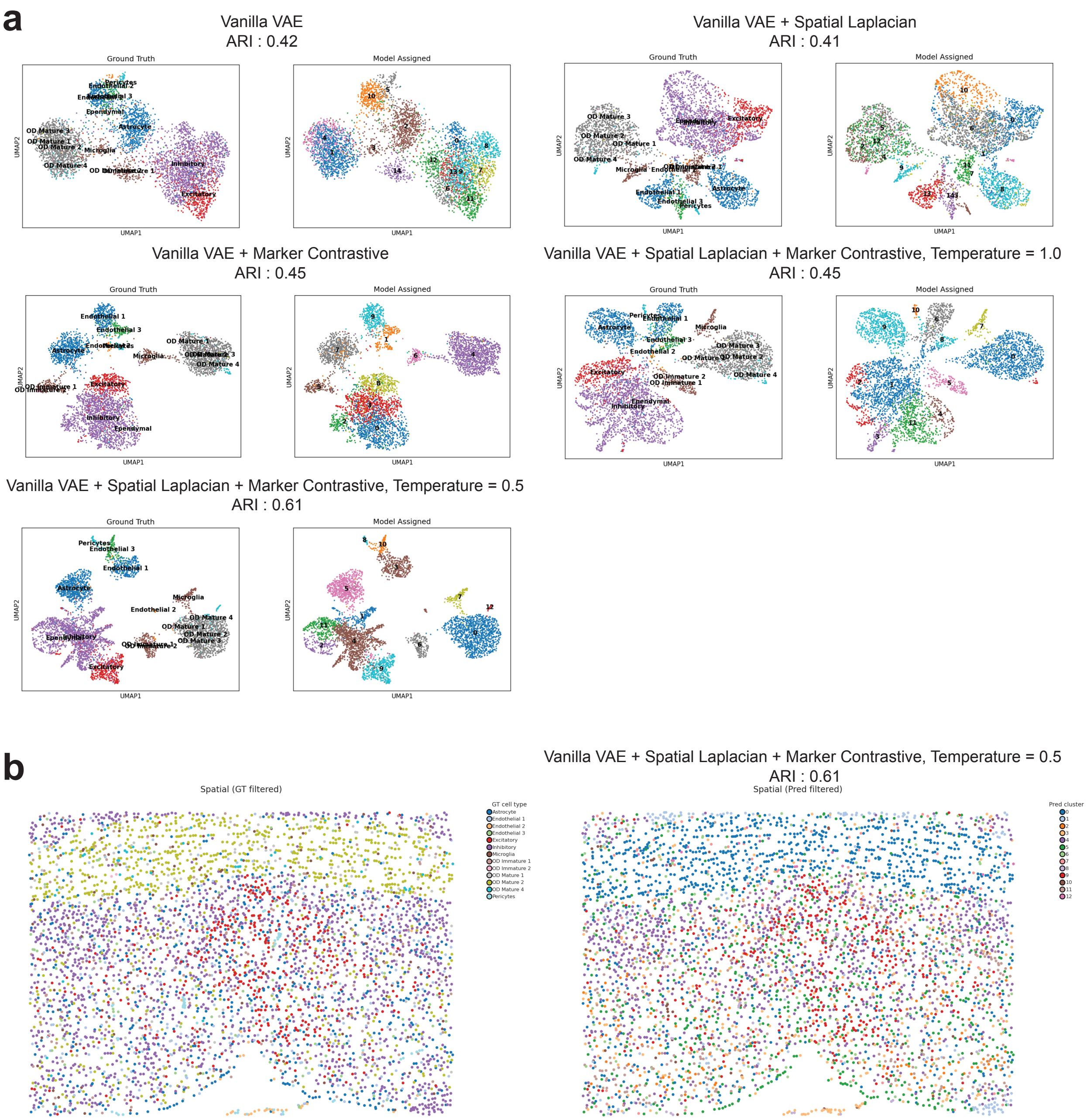

Supplementary Fig. 3: Ablation on MERFISH hypothalamus. **(a)** Progressive inclusion of spatial and biological objectives yields increasingly coherent, anatomically consistent clustering. A vanilla VAE (ARI 0.42) preserves global structure but misses compact nuclei. Adding only contrastive learning (ARI 0.41) sharpens some boundaries but lacks spatial coherence, whereas adding only the Laplacian (ARI 0.45) enforces continuity but merges adjacent types. Joint training requires temperature tuning: high temperature (τ=1.0, ARI 0.47) over-smooths; low temperature (τ=0.1, ARI 0.40) over-segments. The tuned full model (τ=0.5) achieves the best balance (ARI 0.62), recovering compact, anatomically faithful neuronal domains. **(b)** Spatial embeddings (filtered for clusters with $\geq$ 5 cells to exclude small ependymal and mature oligodendrocyte clusters) show that only the full model resolves compact neuronal domains without merging glial boundaries.
